# Antibodies against adenovirus fiber and penton base proteins inhibit adenovirus vector-mediated transduction in the liver following systemic administration

**DOI:** 10.1038/s41598-018-30947-z

**Published:** 2018-08-17

**Authors:** Kyoko Tomita, Fuminori Sakurai, Shunsuke Iizuka, Masahisa Hemmi, Keisaku Wakabayashi, Mitsuhiro Machitani, Masashi Tachibana, Kazufumi Katayama, Haruhiko Kamada, Hiroyuki Mizuguchi

**Affiliations:** 1 Laboratory of Biochemistry and Molecular Biology, Graduate School of Pharmaceutical Sciences, Osaka University, Osaka, Japan; 2 Laboratory of Regulatory Sciences for Oligonucleotide Therapeutics, Clinical Drug Development Unit, Graduate School of Pharmaceutical Sciences, Osaka University, Osaka, Japan; 3 Institute for Frontier Life and Medical Sciences, Kyoto University, Kyoto, Japan; 4 Laboratory of Biopharmaceutical Research, National Institutes of Biomedical Innovation, Health and Nutrition, Osaka, Japan; 5 The Center for Advanced Medical Engineering and Informatics, Osaka University, Osaka, Japan; 6 Laboratory of Hepatocyte Differentiation, National Institute of Biomedical Innovation, Health and Nutrition, Osaka, Japan

## Abstract

Pre-existing anti-adenovirus (Ad) neutralizing antibodies (AdNAbs) are a major barrier in clinical gene therapy using Ad vectors and oncolytic Ads; however, it has not been fully elucidated which Ad capsid protein-specific antibodies are involved in AdNAb-mediated inhibition of Ad infection *in vivo*. In this study, mice possessing antibodies specific for each Ad capsid protein were prepared by intramuscular electroporation of each Ad capsid protein-expressing plasmid. Ad vector-mediated hepatic transduction was efficiently inhibited by more than 100-fold in mice immunized with a fiber protein-expressing plasmid or a penton base-expressing plasmid. An Ad vector pre-coated with FX before administration mediated more than 100-fold lower transduction efficiencies in the liver of warfarinized mice immunized with a fiber protein-expressing plasmid or a penton base-expressing plasmid, compared with those in the liver of warfarinized non-immunized mice. These data suggest that anti-fiber protein and anti-penton base antibodies bind to an Ad vector even though FX has already bound to the hexon, and inhibit Ad vector-mediated transduction. This study provides important clues for the development of a novel Ad vector that can circumvent inhibition with AdNAbs.

## Introduction

Replication-incompetent adenovirus (Ad) vectors are widely used in not only gene therapy studies but also basic research due to their many advantages as a gene delivery vehicle. In addition, recombinant oncolytic adenoviruses (Ads), which efficiently replicate in a tumor cell-specific manner and induce tumor cell death, have gained much attention as a novel antitumor agent. Clinical trials of immunotherapy using an Ad vector expressing foreign antigens and virotherapy using oncolytic Ads against various types of tumors have been ongoing worldwide, and have exhibited promising results^1–4^.

The most crucial concern of gene therapy and virotherapy using recombinant Ads is that pre-existing anti-Ad neutralizing antibodies (AdNAbs) significantly inhibit infection with Ads following not only intravenous administration but also local administration^5,6^. Fifty-seven human Ad serotypes have now been identified and classified into 7 species^7^. Among these serotypes, the most commonly used recombinant Ads, including oncolytic Ads, are based on Ad serotype 5 (Ad5). Previous studies reported the relatively high sero-prevalence of anti-Ad5 antibodies in adults due to natural infection^8–11^. Ad vector-mediated transduction is significantly suppressed in pre-immunized animals. Previous studies reported that AdNAbs mainly recognize three major capsid proteins: the hexon, fiber, and penton base proteins^12–17^. However, it has been highly controversial which Ad capsid protein-specific antibodies are most efficiently produced following Ad infection and exhibit the most efficient inhibition of Ad infection. Inhibitory effects of anti-hexon antibodies on Ad infection have been demonstrated using chimeric Ads containing genetic mutation in the hexon gene^18–20^, while several studies have reported that neutralizing antibodies in human sera and ascetic fluids were primarily directed against the fiber protein and penton base protein^12,21,22^. To resolve this question, it will be necessary to prepare experimental animals possessing antibodies specific for each Ad capsid protein in order to individually and correctly evaluate the effects of antibodies against each capsid protein on Ad vector-mediated *in vivo* transduction. Moreover, in previous studies, inhibition of Ad infection by anti-Ad sera has often been analyzed by determining the *in vitro* transduction efficiencies of Ad vectors in cultured cells in the presence of anti-Ad sera, and by evaluating the vaccination effects following intramuscular administration of Ad vectors encoding foreign antigens in mice pre-immunized with Ads^12,14,16^. Inhibitory effects of anti-Ad capsid protein antibodies on the hepatic transduction with an Ad vector remain to be fully evaluated in spite of the efficient transduction in the liver. It is generally considered that the inhibitory effects of the antibodies of each anti-Ad capsid protein differ according to the target cells and administration routes, and depend on which receptors Ad mainly utilizes for infection of target cells. Commonly used Ads, such as human Ad5, infect cells mainly *via* three pathways—that is, interaction between Ad fiber protein and coxsackievirus-adenovirus receptor (CAR) on the cell surface, interaction between the Arg-Gly-Asp (RGD) motif on the penton base and αv integrins, and interaction between blood coagulation factor X (FX) binding on the hexon and heparan sulfate on the cell surface^23^. FX-dependent infection is more crucial for hepatic transduction with an Ad vector following systemic administration than the other pathways^24^. However, it remains unclear which infection pathway is inhibited by which capsid protein-specific antibodies.

In this study, in order to evaluate in detail the effects of the antibodies of each anti-Ad capsid protein on Ad vector-mediated transduction in the liver, we prepared mice possessing sera of each anti-Ad capsid protein by immunization with a plasmid DNA encoding each Ad capsid protein. Ad vector-mediated transduction in the liver was significantly inhibited in the mice possessing anti-fiber antibodies or anti-penton base antibodies, although anti-fiber protein sera more efficiently inhibited Ad vector-mediated transduction in the liver than anti-penton base sera. In addition, anti-fiber sera inhibited fiber-dependent, penton base-dependent, and FX-dependent transduction with an Ad vector in the cultured cells. Our data suggests that anti-fiber and anti-penton base antibodies play a crucial role in the inhibition of Ad vector-mediated transduction in the liver.

## Results

### Induction of Ad capsid protein-specific antibodies in mice by plasmid DNA electroporation

**Figure 1:**
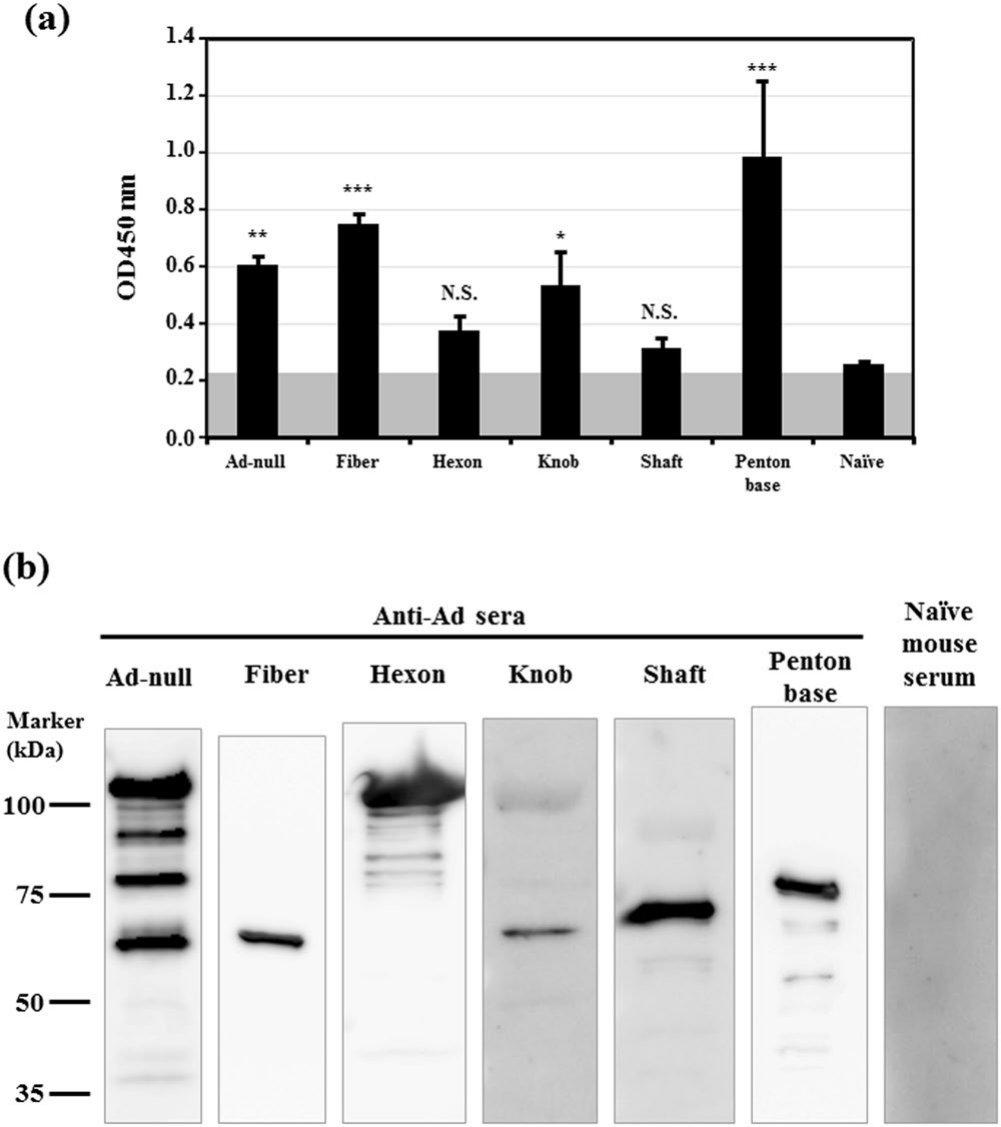
Analysis of Ad capsid protein-specific sera isolated from Ad capsid protein-expressing plasmid-immunized mice. (**A**) ELISA analysis using Ad capsid protein-specific sera. Ad-null was solubilized by 0.1% Triton X-100 and immobilized on a plate. Anti-Ad capsid protein sera were diluted and added to the well. The gray shaded boxes indicate background levels. The data are expressed as the mean ± S.D. (n = 4). n.s., not significant, *p < 0.05, **p < 0.01, ***p < 0.001, compared with naïve sera. (**B**) western blotting analysis using Ad capsid protein-specific sera. Ad-null was denatured at 98 °C for 5 min and loaded on SDS-PAGE gels according to the manufacturer’s protocol. Western blot analysis was carried out using anti-Ad capsid protein sera. Each image was captured under different exposure times. Representative images from three independent experiments using different mouse serum batches are shown.

In order to prepare mice possessing antibodies against each major Ad capsid protein, plasmid DNAs expressing the fiber (full-length fiber protein, fiber knob, and shaft-tail), hexon, or penton base protein were intramuscularly administered, followed by electroporation. Mouse serum was then recovered 2 weeks after the final immunization, followed by an ELISA analysis. A conventional Ad vector containing no transgene expression cassette, Ad-null, was intravenously administered to mice as a control to induce antibodies against various Ad capsid proteins. *In vitro* transfection with major Ad capsid protein-expressing plasmids in HEK293 cells resulted in the production of detectable levels of the corresponding major capsid proteins (Supplementary Fig. 1). An ELISA analysis in which detergent-solubilized Ad proteins were immobilized on the plates demonstrated that the highest titers were found for anti-penton base sera, followed by anti-fiber sera (Fig. 1A). The titers of anti-penton base and anti-fiber sera were comparable to those of anti-Ad capsid protein sera obtained by immunization with Ad-null. Although statistically significant differences were not found between naïve sera, anti-hexon sera, and anti-fiber shaft-tail sera, the averages of antibody titers of anti-fiber shaft-tail sera and anti-hexon sera were higher than those of naïve sera.

Next, to further examine whether anti-Ad capsid protein antibodies were produced by immunization with an Ad capsid protein-expressing plasmid, a western blotting analysis using anti-Ad capsid protein sera was performed. Major capsid proteins, including the hexon (about 108 kDa), penton base (about 68 kDa), and fiber (about 61 kDa) protein, were efficiently detected by the sera of Ad-null-immunized mice, indicating that immunization with Ad-null induced production of antibodies against the major capsid proteins (Fig. 1B). The corresponding Ad capsid proteins were efficiently and specifically detected by the sera of mice immunized with each Ad capsid protein-expressing plasmid. The bands corresponding to the minor capsid proteins VI, VIII, and IX were not clearly detected when the serum samples of Ad-null-immunized mice were used, probably because the titers of anti-minor capsid protein antibodies were lower than those of anti-major capsid protein antibodies. Detectable bands were not found by non-immunized (naïve) mouse serum. These results indicate that each of the major Ad capsid protein-specific antibodies was specifically produced in the mice by immunization with the respective Ad capsid protein-expressing plasmid *via* electroporation in the muscles.

### Transduction efficiencies of an Ad vector in the liver of mice possessing anti-Ad capsid protein sera following intravenous administration

**Figure 2:**
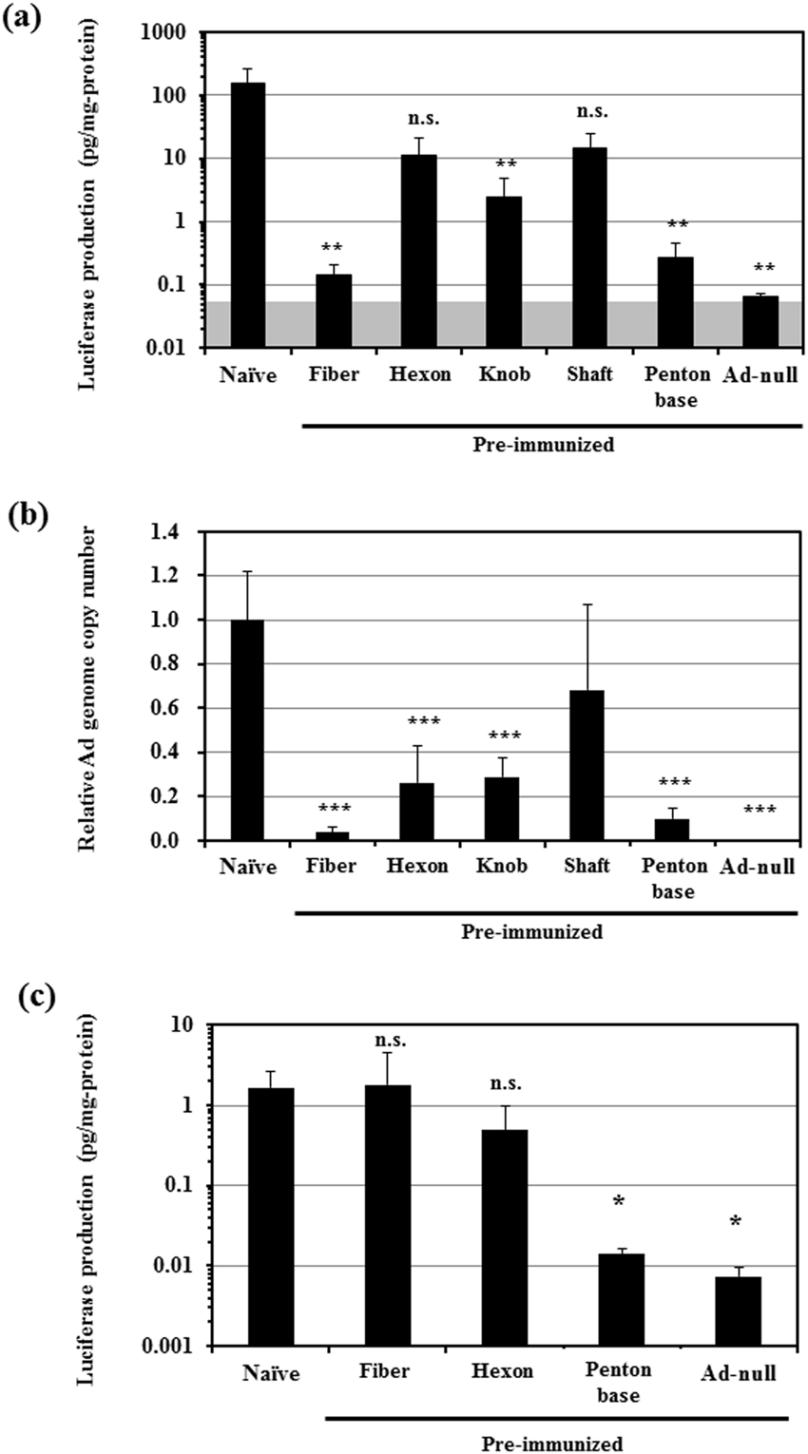
Inhibitory effects of anti-Ad capsid sera on Ad vector-mediated transduction in the liver following intravenous administration in mice immunized with Ad capsid protein-expressing plasmids. (**A**) Transduction efficiencies in the liver following intravenous administration of an Ad vector in the non-immunized and pre-immunized mice. Mice were administered Ad-L2 at a dose of 1 × 10^10^ VP/mouse. The liver was recovered 48 h after administration, followed by luciferase assay. The gray area indicates the background luciferase production levels. (**B**) Ad vector genome copy numbers in the liver following intravenous administration of Ad-L2 in the non-immunized and pre-immunized mice. Mice were administered Ad-L2 as described above. The copy numbers of Ad vector genome in the liver were measured 48 h after administration by real-time PCR analysis. (**C**) Transduction efficiencies of AdF35-L2 in the liver following intravenous administration in mice immunized with Ad capsid protein-expressing plasmids. AdF35-L2 was intravenously administered to mice. Luciferase production levels in the liver were measured 48 h after administration. The data are expressed as the mean ± S.D. (n = 6). n.s., not significant, *p < 0.05, **p < 0.01, ***p < 0.001.

Next, in order to examine the transduction efficiencies of an Ad vector in the liver following systemic administration in mice possessing anti-Ad capsid protein sera, a luciferase-expressing conventional Ad vector, Ad-L2, was intravenously administered to mice immunized with each Ad capsid protein-expressing plasmid. Ad-L2-mediated luciferase expression in the liver was almost completely reduced to the background level in the mice immunized with Ad-null (Fig. 2A). Ad-L2 exhibited about 1000-fold and 500-fold lower transduction efficiencies in the livers of mice possessing anti-fiber and anti-penton base sera, respectively, compared with those in the livers of non-immunized (naïve) mice. Anti-fiber knob serum also significantly inhibited the transduction efficiencies in the liver, although anti-fiber knob serum less efficiently inhibited the hepatic transduction, compared with anti-full-length fiber serum. Anti-fiber shaft-tail and anti-hexon sera did not exhibit statistically significant reduction in the luciferase expression in the liver. These data indicate that antibodies against the fiber and penton base proteins significantly inhibited Ad vector-mediated transduction in the liver.

### Liver accumulation of an Ad vector in the mice possessing anti-Ad capsid protein sera

Next, in order to examine whether antibodies against each Ad capsid protein suppress the liver accumulation of an Ad vector following intravenous administration, the copy numbers of Ad vector genome in the liver were evaluated by real-time PCR analysis. The amounts of Ad vector genome were approximately 26-, 3-, and 10-fold reduced in the livers of mice possessing anti-full-length fiber, anti-fiber knob, and anti-penton base sera, respectively (Fig. 2B). Anti-hexon serum also significantly reduced the Ad vector genome copy numbers in the liver, although the average Ad vector genome copy number in the liver of mice possessing anti-hexon serum was higher than those in the liver of mice possessing anti-fiber and anti-penton base sera. These results indicate that anti-fiber and anti-penton base antibodies efficiently inhibited the liver accumulation of an Ad vector, resulting in a reduction in the hepatic transduction.

We also examined the Ad vector genome copy numbers in the liver and spleen of pre-immunized mice 30 min after Ad vector administration to evaluate whether binding of anti-Ad capsid protein antibodies to Ad vectors promotes the uptake of Ad vectors in liver Kupffer cells and/or spleen macrophages. We previously demonstrated that Ad vector genome taken up by liver Kupffer cells could be detected by recovering the liver within 1 h after administration^25^. Comparable levels of Ad vector genome copy numbers were found in the liver and spleen of naïve mice and mice pre-immunized with Ad capsid protein-expressing plasmids (Supplementary Fig. 2). These results suggested that binding of anti-Ad capsid protein antibodies to Ad vectors did not largely alter the Ad vector uptake in the phagocytic cells in the liver and spleen following administration.

### Transduction with a fiber-substituted Ad vector containing Ad35 fiber protein was not inhibited in mice immunized by an Ad5 fiber protein-expressing plasmid

In order to examine whether anti-Ad5 fiber antibodies specifically inhibited the transduction with an Ad5 vector, we examined the transduction efficiencies of a luciferase-expressing fiber-substituted Ad vector containing the fiber proteins of Ad35 (AdF35-L2)^26^ in the liver of mice immunized with each Ad5 capsid protein-expressing plasmid. Transduction efficiencies of AdF35-L2 were comparable in the livers of naïve mice and mice possessing anti-Ad5 fiber and anti-Ad5 hexon sera, while anti-Ad5 penton base serum significantly inhibited the transduction with AdF35-L2 in the liver by approximately 100-fold (Fig. 2C), probably because the penton base of AdF35-L2 is derived from Ad5. These data indicate that anti-Ad5 fiber antibodies specifically inhibited transduction with an Ad vector containing Ad5 fiber proteins.

### Anti-fiber and anti-penton base sera inhibited FX-dependent transduction in cultured cells

**Figure 3:**
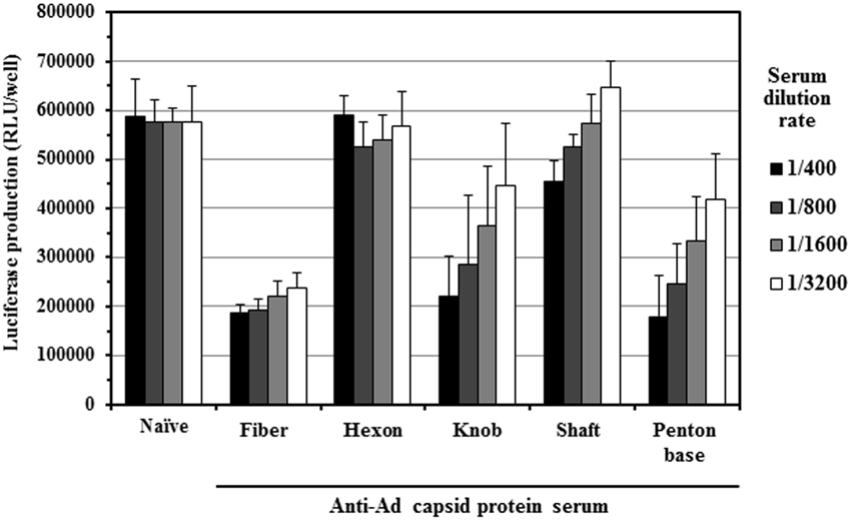
*In vitro* transduction efficiencies of Ad-L2 in CAR-negative cells in the presence of FX and each of the anti-Ad capsid protein sera. LN444 cells were transduced with Ad-L2 at 10000 VP/cell for 1.5 h in the presence of human FX and each anti-Ad capsid protein serum. Luciferase production levels in the cells were determined 24 h after transduction. The data are expressed as the mean ± S.D. (n = 4).

FX binds to the hexon hypervariable regions (HVRs) with high affinity following systemic administration, resulting in efficient transduction in the hepatocytes^9,24^. In order to examine whether anti-fiber and anti-penton base antibodies inhibited the FX-dependent transduction, CAR-negative LN444 cells were transduced with Ad-L2 in the presence of human FX and mouse anti-Ad capsid protein sera. Addition of human FX to the culture media significantly increased the transduction efficiencies of Ad-L2 by more than 150-fold in LN444 cells (Supplementary Fig. 3), indicating that Ad-L2 efficiently transduced LN444 cells in an FX-dependent manner in the presence of FX. As in the case of the transduction in the liver, anti-full-length fiber, anti-fiber knob, and anti-penton base sera significantly reduced the transduction efficiencies by more than 50% in LN444 cells, compared with those in the presence of naïve serum (Fig. 3). The most efficient inhibition was found for anti-full-length fiber serum. Anti-fiber shaft-tail or anti-hexon sera did not show significant inhibition of the *in vitro* transduction in LN444 cells. These results indicated that anti-fiber and anti-penton base antibodies were able to inhibit FX-dependent transduction with an Ad vector in cultured cells.

**Figure 4:**
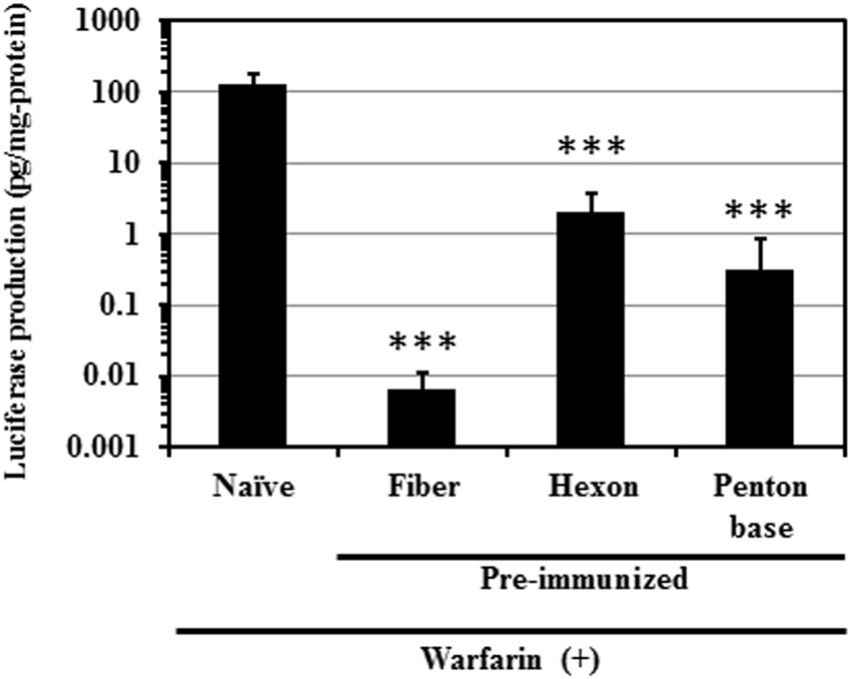
*In vivo* transduction efficiencies of FX-pre-coated Ad-L2 in the liver following intravenous administration in warfarinized mice. Plasmid DNA-immunized and non-immunized mice were warfarinized before administration of an Ad vector. Ad-L2 and FX were mixed and incubated for 30 min before administration. FX-pre-coated Ad-L2 was intravenously administered to mice at a dose of 1 × 10^10^ VP/mouse. Luciferase production levels in the liver were determined 48 h after administration. The data are expressed as the mean ± S.D. (n = 6). ***p < 0.001.

In order to examine whether FX pre-binding on the hexon disturbed the binding of anti-fiber protein and anti-penton base antibodies to Ad vectors, Ad-L2 was pre-incubated with human FX before administration, and subsequently intravenously administered to warfarinized mice pre-immunized with the Ad capsid protein-expressing plasmids. Mice were warfarinized in this experiment to avoid the binding of endogenous mouse FX to Ad capsid proteins before binding of anti-Ad capsid protein antibodies to an Ad vector, and to examine whether anti-Ad capsid protein antibodies were able to access an Ad vector pre-coated with human FX^24,27^. The transduction efficiencies of Ad-L2 without pre-incubation with FX in the liver of normal mice and those of Ad-L2 pre-incubated with FX in the liver of warfarinized mice were comparable (Figs 2A and 4). The previous studies demonstrated that FX binds only to the hexon protein and not to the fiber or penton base proteins^9,24^. We confirmed that FX bound to the Ad particle by a Biacore analysis, and to only the hexon protein by far-western blotting analysis (data not shown). Ad-L2 pre-incubated with human FX mediated approximately 10000-fold lower transduction efficiencies in the liver of mice pre-immunized with a fiber protein-expressing plasmid than those in warfarinized mice without pre-immunization (Fig. 4). Anti-penton base serum also significantly inhibited the transduction with Ad-L2 pre-incubated with FX in the liver of warfarinized mice. Statistically significant reduction in the transduction efficiencies of Ad-L2 was induced by anti-hexon serum under this experimental condition, although the average of transduction efficiencies in the liver of warfarinized mice possessing anti-hexon antibodies were higher than those in the liver of warfarinized mice possessing anti-fiber antibodies. These results indicated that anti-fiber protein and anti-penton base antibodies bound to an FX-coated Ad vector, leading to the inhibition of hepatic transduction with an Ad vector.

### Anti-fiber and anti-penton base sera inhibited CAR-dependent and αv integrin-dependent transduction

**Figure 5:**
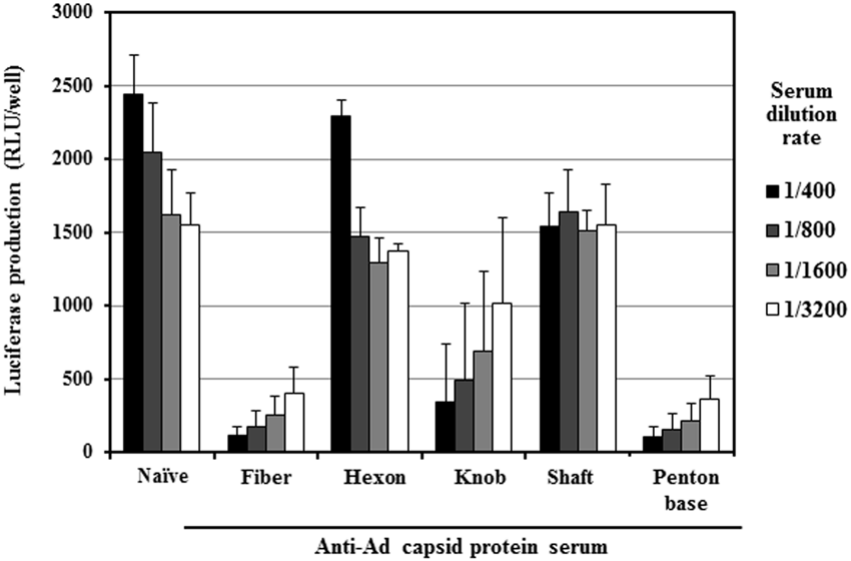
*In vitro* transduction efficiencies of Ad-L2 in CAR-positive cells by each of the anti-Ad capsid protein sera. A549 cells were transduced with Ad-L2 at 500 VP/cell for 1.5 h in the presence of each anti-Ad capsid protein serum. Luciferase production levels in the cells were determined 24 h after transduction. The data are expressed as the mean ± S.D. (n = 4).

Several studies, including ours, have demonstrated that the ablation of both CAR- and αv integrin-binding from an Ad vector significantly reduced the transduction efficiencies in the liver, indicating that CAR- and αv integrin-binding are also involved in Ad vector-mediated transduction in the liver^28–31^. In order to examine whether anti-fiber and anti-penton base antibodies inhibit the CAR- and/or αv integrin-dependent transduction, CAR- and αv integrin-positive A549 cells were transduced with Ad-L2 in the presence of anti-Ad capsid protein sera. Transduction efficiencies of Ad-L2 gradually decreased as the dilution factors of naïve serum increased, probably because mouse FX remaining in the serum samples of naïve mice contributed to Ad vector-mediated transduction in the cultured cells (Fig. 5). Transduction with Ad-L2 was significantly suppressed in the presence of anti-full-length fiber, anti-fiber knob, and anti-penton base sera. On the other hand, no apparent reduction in the transduction efficiencies was observed in the presence of anti-hexon serum, compared with naïve serum. These results suggested that CAR- and/or αv integrin-dependent transduction in cultured cells was inhibited by anti-fiber and anti-penton base antibodies.

**Figure 6:**
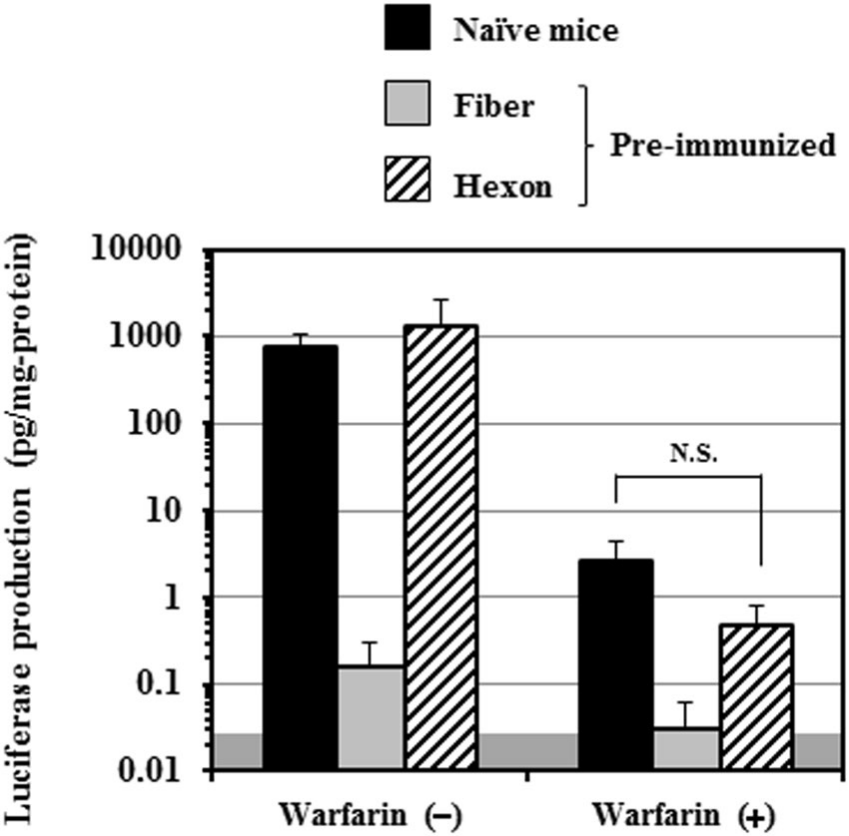
*In vivo* transduction efficiencies of Ad-L2 in the liver of warfarinized mice following intravenous administration. Plasmid DNA-immunized and non-immunized mice were warfarinized before administration of an Ad vector. Ad-L2 was intravenously administered to the mice at a dose of 1 × 10^10^ VP/mouse. The liver was recovered 48 h after administration, followed by luciferase assay. The gray area indicates the background luciferase production levels. The data are expressed as the mean ± S.D. (n = 6). n.s., not significant.

Next, in order to further examine the inhibitory effects of anti-fiber serum on CAR-dependent and αv integrin-dependent transduction in the liver, mice pre-immunized with Ad capsid protein-expressing plasmids were warfarinized, followed by intravenous administration of an Ad vector. An approximately 300-fold reduction in the transduction efficiencies was found in the liver of warfarinized mice, compared with that of non-warfarinized mice (Fig. 6), as previously reported^24,32^. Anti-hexon serum failed to mediate statistically significant inhibition in the liver transduction in the warfarinized mice. Immunization with a fiber protein-expressing plasmid resulted in a significant reduction in the transduction efficiencies in the liver of warfarinized mice. These results suggest that anti-fiber antibodies inhibited CAR- and/or αv integrin-dependent transduction in the liver.

### Anti-fiber serum inhibited transduction with CAR-binding ablated and αv integrin-binding ablated Ad vectors

**Figure 7:**
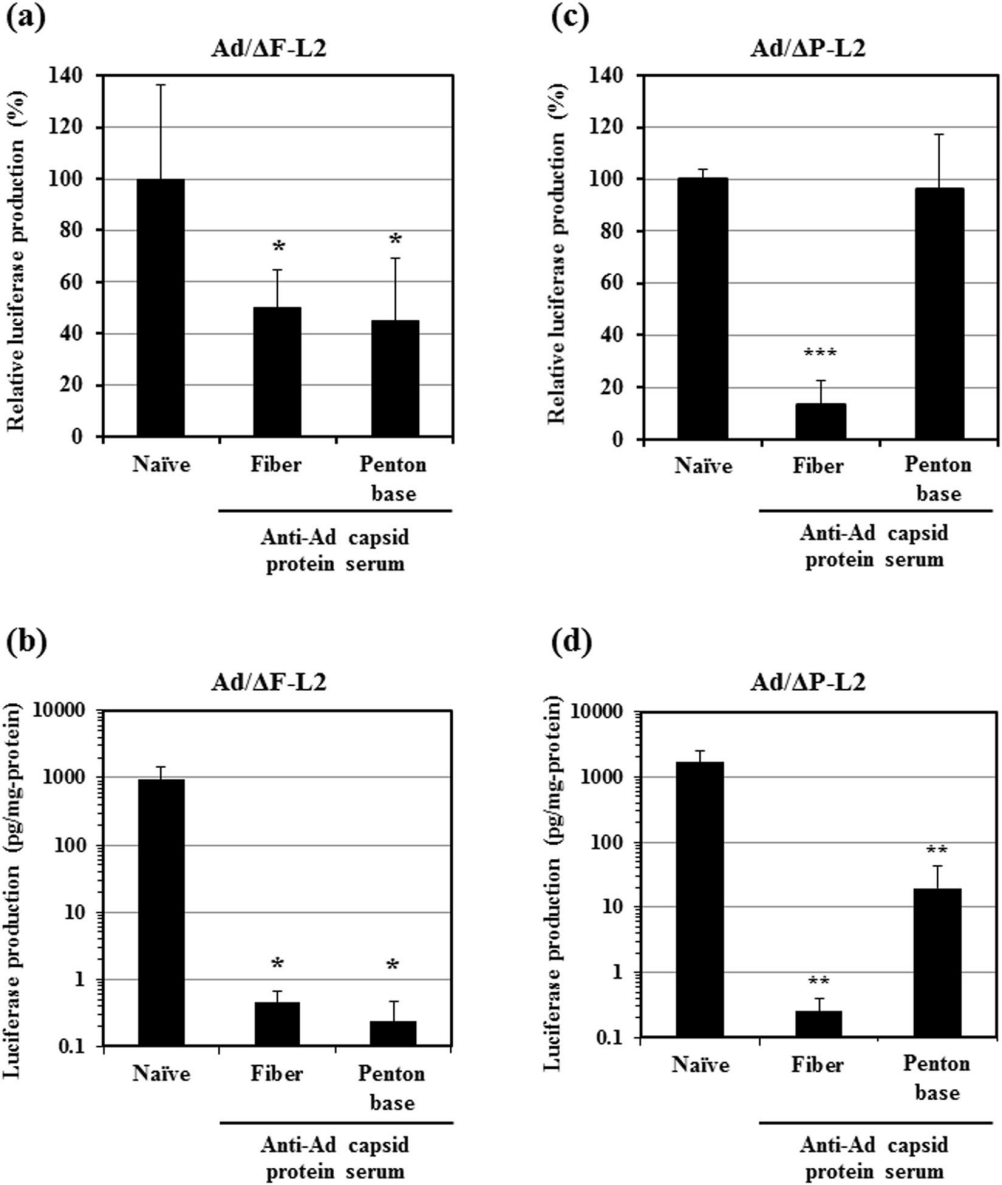
Anti-Ad capsid protein serum-mediated inhibition of transduction efficiencies of Ad/ΔF-L2 and Ad/ΔP-L2. (**A**,**C**) *In vitro* transduction efficiencies of Ad/ΔF-L2 and Ad/ΔP-L2 in A549 cells in the presence of each of the anti-Ad capsid protein sera. A549 cells were transduced with Ad-L2 at 500 VP/cell for 1.5 h in the presence of each anti-Ad capsid protein serum. Luciferase production levels in the cells were determined 24 h after transduction. The data are expressed as the mean ± S.D. (n = 4). (**B**,**D**) *In vivo* transduction efficiencies of Ad/ΔF-L2 and Ad/ΔP-L2 in the liver of pre-immunized mice following intravenous administration. Ad-L2 was administered to the pre-immunized mice at a dose of 1 × 10^10^ VP/mouse. The liver was recovered 48 h after administration, followed by luciferase assay. The gray area indicates the background luciferase production levels. The data are expressed as the mean ± S.D. (n = 6). *p < 0.05, **p < 0.01, ***p < 0.001.

We further examined the inhibitory effects of anti-Ad capsid protein antibodies on CAR-dependent and αv integrin-dependent transduction by using CAR-binding ablated Ad vector (Ad/ΔF-L2)^28^ and αv integrin-binding ablated Ad vector (Ad/ΔP-L2)^28^. Ad/ΔF-L2 and Ad/ΔP-L2 possess genetic mutation in the domain crucial for CAR-binding in the fiber knob and the RGD motif in the penton base, respectively. We examined the transduction efficiencies of Ad/ΔF-L2 and Ad/ΔP-L2 in the presence of anti-fiber and anti-penton base sera in A549 cells and the liver of pre-immunized mice. Transduction with Ad/ΔF-L2 was significantly inhibited by more than 50% in the presence of anti-fiber and anti-penton base sera in A549 cells (Fig. 7A). Transduction efficiencies of Ad/ΔF-L2 in the liver were also largely reduced in the mice pre-immunized with fiber protein-expressing and penton base-expressing plasmids (Fig. 7B). Transduction with Ad/ΔF-L2 in cultured cells is considered to be mainly mediated by interaction between the RGD motif in the penton base and αv integrins on the cells. These data suggest that not only anti-penton base antibodies but also anti-fiber antibodies inhibited the transduction through the interaction between the penton base and αv integrins.

Transduction efficiencies of Ad/ΔP-L2 in A549 cells were also significantly reduced in the presence of anti-fiber serum, while Ad/ΔP-L2 mediated comparable levels of transduction efficiencies in the presence and absence of anti-penton base serum (Fig. 7C). Ad/ΔP-L2 is considered to mediate transduction mainly *via* interaction between the fiber knob and CAR on the cell surface in cultured cells. These data suggest that anti-fiber antibodies, not anti-penton base antibodies, disturbed the transduction *via* the interaction between the fiber knob and CAR. The livers of mice pre-immunized with a fiber protein-expressing plasmid also exhibited a significant reduction in the transduction efficiencies of Ad/ΔP-L2 (Fig. 7D). Although anti-penton base serum also significantly inhibited transduction with Ad/ΔP-L2 in the liver, anti-penton base serum less efficiently reduced the transduction efficiencies of Ad/ΔP-L2 in the liver than anti-fiber serum. Anti-penton base antibodies produced in mice by immunization with a penton base-expressing plasmid might have less efficiently recognized the penton base of Ad/ΔP-L2 due to the genetic mutation in the penton base.

## Discussion

The aim of this study was to reveal the effects of anti-Ad capsid protein antibodies on Ad vector-mediated transduction in the liver. It has remained a subject of controversy which Ad capsid protein-specific antibodies play an important role in the inhibition of Ad vector-mediated transduction, partly because experimental animals have often been immunized with Ad whole virions, not individual Ad capsid proteins, in the previous studies^14,16,33^. Antibodies against almost all the Ad capsid proteins are produced in the animals immunized with Ad virions. It is difficult to individually and correctly evaluate the effects of each anti-Ad capsid protein antibody when Ad virions are used for immunization. In this study, mice were immunized with each Ad capsid protein-expressing plasmid *via* intramuscular electroporation, resulting in efficient induction of each anti-Ad capsid protein antibody.

Ad vector-mediated transduction in the liver was efficiently inhibited in the mice possessing anti-fiber serum (Fig. 2). Anti-fiber serum also inhibited FX-dependent *in vitro* transduction in CAR-negative cells (Fig. 3). In addition, anti-fiber serum significantly inhibited liver transduction with an FX-pre-coated Ad vector in warfarinized mice (Fig. 4). These findings suggest that anti-fiber antibodies bind to the fiber protein even though FX has already bound to the hexon, and inhibit interaction between FX and heparan sulfate on the cell surface. The molecular weights of FX, fiber protein, and IgG are 48 kDa, 61 kDa, and 150 kDa, respectively. Anti-fiber antibodies would inhibit the interaction between FX and heparan sulfate *via* steric hindrance. Generally, binding of antibodies to virus promotes Fc receptor-mediated uptake in liver Kupffer cells and spleen macrophages, but the copy numbers of Ad vector genome in the liver and spleen of mice pre-immunized with Ad capsid protein-expressing plasmids and naïve mice were comparable 30 min after administration (Supplementary Fig. 2). In addition, as described above, *in vitro* transduction in CAR-negative cells in the presence of FX showed that anti-fiber protein antibodies significantly inhibited the FX-dependent transduction with an Ad vector in the cells (Fig. 3). The significant reduction in the transduction efficiencies in the liver of mice pre-immunized with the fiber protein-expressing plasmid would not be solely due to anti-fiber antibody-mediated enhancement of Ad vector uptake in the liver Kupffer cells and spleen macrophages.

Waddington *et al*. demonstrated that the KD value of FX-Ad interaction was approximately 1.8 × 10^−9^ M^24^, although the KD value of antigen-antibody interaction is generally reported as 10^−8^-10^−10^ M^34,35^. These data indicated that the affinity of FX-Ad interaction was comparable to that of antigen-antibody interaction and that FX-Ad interaction was stable in the blood. Recently, Harmon *et al*. reported that human FX inhibits the neutralization of Ad5 vectors by human serum^36^, suggesting that FX binding to the hexon would prevent anti-hexon antibody-mediated neutralization.

In addition to the interaction between FX on the hexon and heparan sulfate on the cell surface, binding of fiber knob to CAR and RGD motif in the penton base to αv integrins are also involved in Ad vector-mediated *in vivo* transduction in the liver. Ablation of both CAR- and αv integrin-binding in Ad vectors significantly reduced the transduction efficiencies in the liver following intravenous administration^29,30^. This study demonstrated that anti-fiber serum significantly inhibited the *in vitro* transduction with Ad/ΔF-L2 and Ad/ΔP-L2 in CAR-positive A549 cells (Fig. 7). In addition, transduction efficiencies in the liver were also reduced in the warfarinized mice pre-immunized with a fiber protein-expressing plasmid (Fig. 6). These results indicate that both CAR-dependent and αv integrin-dependent transductions are inhibited by anti-fiber antibodies.

Anti-penton base serum also efficiently inhibited the transduction in the liver (Fig. 2). FX-dependent *in vitro* transduction in CAR-negative cells was inhibited by anti-penton base serum (Fig. 3). These results indicate that anti-penton base antibodies inhibit FX-dependent transduction in the liver following systemic administration. In addition, anti-penton base serum inhibited αv integrin-dependent transduction in cultured cells (Fig. 7A). However, overall, the inhibitory effects of anti-penton base serum on Ad vector-mediated transduction seemed less efficient, compared with those of anti-fiber serum, despite the fact that the antibody titers of anti-penton base sera were higher than those of anti-fiber sera in an ELISA analysis (Fig. 1A). CAR-dependent transduction was not disturbed by anti-penton base serum (Fig. 7C). Anti-fiber antibodies thus appear to play a more important role in AdNAb-mediated inhibition of transduction with an Ad vector than anti-penton base antibodies.

The findings in this study provide important clues for modification of Ad vectors to circumvent the inhibition of Ad vector-mediated transduction by AdNAbs. Genetic modification of fiber proteins and penton base makes it possible to escape from inhibition by AdNAbs. Various types of Ad vectors containing fiber proteins derived from other serotype Ads have been developed mainly for re-targeting to CAR-negative cells^26,37–39^. Several studies have demonstrated that inhibition by AdNAbs can be partially bypassed by fiber substitution^15,40,41^, although we should exercise caution in regard to potential cross-reactivities of anti-fiber antibodies with fiber proteins of different Ad serotypes.

One major concern about immunization with an Ad capsid protein-expressing plasmid is that the profiles of anti-Ad capsid protein antibodies, including epitopes and immunoglobulin classes, differ among natural infection with Ads, immunization with Ad virions, and immunization with an Ad capsid protein-expressing plasmid. A previous study demonstrated that anti-fiber antibodies were mainly induced following natural infection, whereas antibodies primarily directed to capsid proteins other than fiber proteins were elicited following Ad vector vaccination *via* intramuscular administration in clinical trials^42^. Perhaps epitopes of anti-fiber antibodies induced by a fiber protein-expressing plasmid in this study might be different from those following natural infection with Ads; however, fiber protein was efficiently detected by the anti-fiber serum induced by intramuscular administration of a fiber-expressing plasmid in an ELISA analysis (Fig. 1A), suggesting that the anti-fiber antibodies induced by a fiber-expressing plasmid recognized the native form of fiber protein. Further analysis will be needed to reveal the differences in the profiles of AdNAbs following natural infection and immunization with recombinant Ads and plasmids.

Previous studies reported that anti-hexon antibodies were efficiently induced following Ad infection and contributed to inhibition of Ad vector-mediated transduction by AdNAbs^14,16^. Genetic mutation of hypervariable regions (HVRs) in the hexon made it possible to circumvent the inhibitory effects of AdNAbs following intramuscular administration in mice^13,16,19,33^. HVRs contain major neutralizing determinants. In addition, interaction between FX on the hexon and heparan sulfate on the hepatocytes is considered to play a crucial role in Ad vector-mediated transduction in the liver^9,24^. FX mainly binds to the hypervariable region (HVR)5 in the hexon^9^. These findings led us to hypothesize at the beginning of this study that Ad vector-mediated transduction in the liver would be most efficiently inhibited by anti-hexon antibodies. However, significant inhibition of liver transduction with an Ad vector was not found in mice immunized with a hexon-expressing plasmid. It thus remains to be clarified why Ad vector-mediated transduction in the liver was not suppressed in mice possessing anti-hexon serum in this study. In the previous studies showing that anti-hexon antibodies mainly contributed to the inhibition of Ad vector-mediated transduction^18–20^, the titers of anti-hexon antibodies may have been much higher than those in this study. In this study, a clear band corresponding to hexon protein was detected by anti-hexon sera in the western blotting analysis (Fig. 1B), indicating that anti-hexon antibodies were produced by immunization with a hexon-expressing plasmid; however, statistically significant differences in anti-Ad capsid protein antibody titers were not found between anti-hexon sera and naïve sera in the ELISA analysis (Fig. 1A). In addition, the titers of anti-hexon antibodies were lower than those of anti-fiber and anti-penton base antibodies. Otherwise, as described above, the epitopes of anti-hexon antibodies may differ greatly between immunization with a hexon-expressing plasmid and immunization with Ad virions.

A hexon-expressing plasmid was used for induction of anti-hexon antibodies in this study. We confirmed the hexon protein production following transfection in HEK293 cells (Supplementary Fig. 1). A previous study reported that detectable levels of hexon protein were not found by western blotting analysis following transfection with a hexon-expressing plasmid alone in cells of the monkey kidney fibroblast line COS-7, but hexon protein was efficiently produced when the virus-encoded 100 K protein was co-expressed^43^. Hexon trimer assembly required co-expression of the virus-encoded 100 K protein^44^. The differences in the hexon expression levels between this study and the previous study were probably due to the differences in the experimental conditions, including the transfection reagents, cell lines, plasmid vectors, and sensitivities of chemiluminescence in western blotting analysis.

In summary, we evaluated the inhibitory effects of anti-Ad capsid sera on hepatic transduction with an Ad vector following systemic administration using mice immunized with an Ad capsid protein-expressing plasmid. Anti-fiber and anti-penton base sera significantly inhibited the transduction in the liver. These findings provide important clues for development of a novel Ad vector and oncolytic Ad that can circumvent inhibition by anti-Ad neutralizing antibodies.

## Materials and Methods

### Cell culture

A549 cells (a human lung adenocarcinoma epithelial cell line), HEK293 cells (a human embryonic kidney cell line), and LN444 cells (a human glioma cell line) were cultured in Dulbecco’s modified Eagle’s medium (DMEM) (Wako Pure Chemical Industries, Osaka, Japan) supplemented with 10% fetal bovine serum (FBS) and antibiotics.

### Plasmids and Ad vectors

A plasmid DNA expressing each Ad5 capsid protein was constructed as follows. First, a fragment encoding major Ad capsid proteins was amplified by PCR and cloned into the multicloning sites of pCMV-SL3^45^. The penton base, shaft, and knob protein were FLAG-tagged. An Ad vector containing no transgene expression cassette (Ad-null) was previously constructed^46^. A luciferase-expressing conventional Ad vector, Ad-L2, a fiber-substituted luciferase-expressing Ad vector containing the fiber protein of Ad serotype 35 (Ad35), AdF35-L2, and CAR binding-ablated and αv integrin binding-ablated Ad vectors expressing luciferase, Ad/ΔF-L2 and Ad/ΔP-L2, respectively, were previously constructed^26,28,37^. Determination of the virus particle (VP) titers was accomplished spectrophotometrically by the method of Maizel *et al*.^47^.

### Immunization with Ad capsid protein-expressing plasmids and an Ad vector

C57BL/6 J mice (6–8 weeks-old, female) were purchased from Japan SLC (Hamamatsu, Japan). Mice were intramuscularly immunized twice with a plasmid DNA expressing each Ad capsid protein in the right and left quadriceps muscles (50 μg per muscle; total 100 μg per mouse) at 0 and 4 weeks by *in vivo* electroporation (e.p.) as previously described^48^. For immunization of mice with an Ad vector, mice were intravascularly administered Ad-null at a dose of 1 × 10^10^ VP/mouse *via* the tail vein. The production of antibodies against each Ad capsid protein in the serum was evaluated at 6 weeks after the first immunization (see below). Pre-immunized mice were used for transduction experiments 6 weeks after the first immunization. All procedures involving animals and their care were approved by the Institutional Animal Care and Use Committees of National Institute of Biomedical Innovation, Health and Nutrition.

### Western blotting analysis

Serum samples were collected from mice by retro-orbital bleeding. Ad protein samples were prepared by incubation of 1 × 10^10^ VP of Ad-null with sample buffer (250 mM Tris-HCl pH 6.8, 30% 2-mercaptoethanol, 10% sodium dodecyl sulfate (SDS), 20% glycerol) and denatured at 98 °C for 5 min. Ad protein samples were then electrophoresed on 12.5% SDS-polyacrylamide gels (Wako Pure Chemical Industries) according to the manufacturer’s protocol, followed by electrotransfer to PVDF membranes (Millipore, Bedford, MA). After blocking with Immunoblock (DS Pharma, Osaka, Japan), the membrane was incubated with mouse sera, followed by incubation in the presence of horseradish peroxidase (HRP)-labeled anti-mouse IgG antibody (Cell Signaling Technology, Danvers, MA). Dilution factors of mouse sera were 1:100–1000. Protein bands of the membrane were visualized with a chemiluminescence kit (Chemi-Lumi One Super; Nacalai Tesque, Kyoto, Japan).

### ELISA analysis of anti-Ad capsid protein antibodies

ELISA analysis of anti-Ad capsid protein sera was performed as previously described^49^. Briefly, Ad capsid proteins were solubilized by vortexing of Ad-null in the presence of 0.1% triton-X solution for 10 min. Solubilized Ad capsid proteins were immobilized on a 96-well plate at a density of 5 × 10^6^ VP/well after 100-fold dilution by carbonate buffer. Anti-Ad capsid protein sera were 400-fold diluted by PBST containing Immunoblock, and were added to the well, followed by a 2-h incubation at 37 °C. The plates were then washed with PBST and incubated with biotin-labeled goat anti-mouse IgG antibody (SouthernBiotech, Birmingham, AL) for a 2-h incubation at 37 °C. After washing, streptavidin-HRP (SouthernBiotech) was added to the well, followed by a 1-h incubation at room temperature. Finally, TMB ELISA Peroxidase Substrate (Rockland Immunochemicals, Gilbertsville, PA) was added. The reaction was stopped by the addition of 0.5 mol/l HCl, and absorbance was read at 450 nm on a TriStar LB941 multimode reader (Berthold Technologies, Bad Wildbad, Germany).

### Ad vector-mediated transduction in mice

Mice were intravenously administered Ad-L2 at a dose of 1 × 10^10^ VP/mouse, or AdF35-Luc at a dose of 5 × 10^10^ VP/mouse, *via* the tail vein. Two days following administration, the livers were recovered and homogenated as previously described^50^. Luciferase production levels were determined using a luciferase assay system (PicaGene 5500; Toyo Inki Co., Tokyo, Japan). For warfarinization of mice, mice were subcutaneously injected with warfarin (Cayman Chemical, Ann Arbor, MI) dissolved in 100 uL of peanut oil at a dose of 133 μg per mouse 3 days and 1 day prior to i.v. injection of Ad vectors. As a control, 100 μL of peanut oil was injected. Ad-L2 (1 × 10^10^ VP/mouse) were mixed with human FX (10 μg/mouse) and incubated at room temperature for 30 min before administration, and then the mixture was intravenously administered to mice *via* the tail vein. Luciferase production levels in the liver were determined 48 h after Ad vector administration as described above.

### Real-Time PCR analysis of Ad vector genome copy numbers

Total DNA, including the Ad vector genome, was isolated from the liver by an automatic nucleic acid isolation system (NA-2000; Kurabo Industries, Osaka, Japan) 48 h after administration of Ad vectors. The copy numbers of Ad genomic DNA in the liver were quantified with the TaqMan fluorogenic detection system (StepOnePlus^TM^ Real Time PCR System; Applied Biosystems, Foster City, CA) as previously described^51^.

### Ad vector-mediated transduction in cultured cells

Inhibitory effects of antibodies against each Ad capsid protein in the serum on transduction in cultured cells were performed as follows. Briefly, cells were seeded on a 96-well plate at 1 × 10^4^ cells/well. On the following day, mouse anti-Ad capsid protein sera were serially diluted, and added to each well. Naïve mouse serum was used as a control. Mouse anti-Ad capsid protein sera were not pooled. The mouse serum isolated from each mouse was separately tested in each dilution series. Cells were then transduced with Ad-L2 at 500 VP/cell for 1.5 h. The final dilution factors of mouse anti-Ad capsid protein sera were from 1/400 to 1/3200. After a 24 h-incubation, luciferase production was determined using a luciferase assay system (PicaGene LT2.0; Toyo Inki Co.). For evaluation of transduction efficiencies in CAR-negative cells in the presence of human FX, LN444 cells were seeded on a 96-well plate at 1 × 10^4^ cells/well. On the following day, human FX at a final concentration of 2 μg/mL and mouse anti-Ad capsid protein sera were added to each well as described. Cells were transduced with Ad-L2 at 10000 VP/cell for 1.5 h. After a 24-h incubation, luciferase production was determined using a luciferase assay system. In the experiments using Ad/ΔF-L2 and Ad/ΔP-L2, A549 cells were transduced with Ad/ΔF-L2 at 3000 VP/cell or Ad/ΔP-L2 at 500 VP/cell. Luciferase production was determined as described above.

### Statistical analysis

Statistical significance was determined using one-way Anova followed by Dunnett test. Data are presented as the mean ± S.D.

## Electronic supplementary material

Supplementary Figures
