# Supplementary material for: Antibodies against adenovirus fiber and penton base proteins inhibit adenovirus vector-mediated transduction in the liver following systemic administration
Source: Sci Rep. 2018 Aug 17;8:12315. doi: 10.1038/s41598-018-30947-z (PMC6098129; doi:10.1038/s41598-018-30947-z)
Supplement: Supplementary file 1 — Supplementary Figures [file 41598_2018_30947_MOESM1_ESM.docx]

Antibodies against adenovirus fiber and penton base proteins inhibit adenovirus vector-mediated transduction in the liver following systemic administration.

Kyoko Tomita^1,#^, Fuminori Sakurai^1,2,#,^*, Shunsuke Iizuka^1^, Masahisa Hemmi^1^, Keisaku Wakabayashi^1^, Mitsuhiro Machitani^1,3^, Masashi Tachibana^1^, Kazufumi Katayama^1^, Haruhiko Kamada^4,5^, Hiroyuki Mizuguchi^1,5,6,^*

**Supplementary Figure 1**

Major Ad capsid protein expression in HEK293 cells following transfection with a plasmid expressing each major Ad capsid protein. Total protein lysates were prepared 72 h after transfection, followed by western blotting analysis. The experiments were repeated at least twice. Representative images are shown.

**Supplementary Figure 2**

Ad vector genome copy numbers in the liver and spleen 30 min after intravenous administration of Ad-L2 in the non-immunized and pre-immunized mice. Mice were administered Ad-L2 at a dose of 1x10^10^ VP/mouse. The organs were recovered 30 min after administration, followed by total DNA isolation from the organs and real-time PCR analysis. The data are expressed as the mean± S.E. (n=3-4).

**Supplementary Figure 3**

Transduction efficiencies of Ad-L2 in CAR-negative cells in the presence or absence of FX. LN444 cells were transduced with Ad-L2 at 10000 VP/cell for 1.5 h in the presence or absence of FX. Luciferase production levels were determined 24 h after transduction. The data are expressed as the mean± S.D. (n=4).
